# Thromboembolic disease and hemostatic alterations in tumor-bearing dogs – A narrative review

**DOI:** 10.3389/fvets.2026.1818630

**Published:** 2026-06-05

**Authors:** Paolo Pazzi, Annemarie T. Kristensen

**Affiliations:** 1 Department of Small Animal Clinical Sciences, College of Veterinary Medicine, University of Tennessee, Knoxville, TN, United States; 2 Department of Veterinary Clinical Sciences, Faculty of Health and Medical Sciences, University of Copenhagen, Copenhagen, Denmark

**Keywords:** cancer, coagulation, emboli, hypercoagulable, thrombus, lymphoma, carcinoma, sarcoma

## Abstract

Hemostatic dysfunction is a prevalent and significant complication in tumor-bearing dogs, driven by complex interactions between the tumor microenvironment and the host's coagulation system. This narrative review examines primary and secondary hemostatic alterations, fibrinolytic abnormalities, and prognostic indicators associated with hemostatic variables in canine lymphoma, carcinoma, and sarcoma. In lymphoma, the true prevalence of thromboembolic disease (TED) is unknown; clinically significant hemostatic alterations are typically associated with advanced stages, characterized by persistent hypercoagulability, or elevated D-dimer concentrations, and may be associated with a poor prognosis. Carcinomas are associated with TED, and hemostatic alterations are common, typically characterized by reactive thrombocytosis, hyperfibrinogenemia, and viscoelastic evidence of hypercoagulability. Sarcomas appear to be most associated with TED. Soft tissue sarcoma and osteosarcoma exhibit different hemostatic alterations and prognostic variables compared with hemangiosarcoma, and these differences are typically related to the stage of the disease. By synthesizing current evidence, recent advances, and key knowledge gaps, this review identifies the need to define tumor-specific hemostatic diagnostic profiles in tumor-bearing dogs and subsequently develop tailored thromboprophylaxis strategies. This may ultimately improve survival by enabling targeted management of hemostatic alterations and, potentially, by mitigating metastatic progression.

## 1 Introduction

In human oncology, up to 10%−20% of patients experience clinically apparent venous thromboembolism, making thromboembolic events the second leading cause of death in cancer patients (1, 2). Laboratory evidence of hemostatic alterations is reported in 58%−98% of human oncology cases (3). In tumor-bearing dogs, hemostatic dysfunction is reported to range between 57 and 67% (4, 5). Affected dogs may exhibit a wide range of hemostasis alterations, from mild laboratory hemostatic abnormalities consistent with hypercoagulability and non-overt disseminated intravascular coagulation (DIC), to clinically evident thrombosis or overt DIC with associated hypocoagulability and severe bleeding (5, 6), contributing to morbidity and complicating patient management (7). Non-overt DIC has been documented in carcinomas and sarcomas, with or without metastasis (5, 6). Overt DIC has been documented most commonly in dogs with advanced metastatic tumors such as hemangiosarcoma (HSA), inflammatory mammary carcinoma, and acute leukemia (4, 8, 9). Macroscopic thromboembolic disease (TED) of the pulmonary, portal, and splenic vasculature, as well as in the aorta and cranial vena cava has been associated with tumors in dogs (10–16). The 2019 CURATIVE guidelines consider only a subset of dogs with (adeno)carcinomas at risk of TED from tumors (17), but hemostatic dysfunction is highly prevalent and may contribute to tumor progression and metastasis while also complicating patient management (6, 7, 18, 19).

Studies detailed in this review used various laboratory assays of plasma-based hemostatic variables, including prothrombin time (PT), activated partial thromboplastin time (aPTT), fibrinogen concentration, specific factor (V, VII, VIII, IX, X, XI, XII) activity, antithrombin (AT) activity, thrombin-AT complexes, D-dimer (D-dimer) concentration, fibrinogen degradation products (FDPs), plasminogen concentration, plasminogen activator inhibitor-1 (PAI-1), serum urokinase-type plasminogen activator. Reported variations from normal were defined by comparison to healthy controls or by reference intervals specific to the laboratory or institution. Additionally, various viscoelastic modalities (e.g., rotational thromboelastometry (ROTEM) or thromboelastography (TEG)) with different activators (TF or kaolin) or modifiers (tissue plasminogen (tPA)) have been reported, each with institution-specific reference intervals or study-specific control groups used for comparison. No clear guidelines exist for defining hypercoagulability or hypocoagulability in veterinary medicine. Using TEG as an example, the term hypercoagulable (e.g., decreased R or K time, increased angle, MA or G) or hypocoagulable (e.g., increased R or K time, decreased angle, MA or G) as used in this review reflects the criteria used by each study to define the state based on viscoelastic testing, with variables from other platforms (e.g., ROTEM or VCM) interpreted using the terminology specific to each system.

This review highlights the pathophysiology underlying hemostatic abnormalities in tumor-bearing dogs. It then describes and evaluates tumor-associated TED, primary and secondary hemostatic alterations, abnormalities in fibrinolysis, as well as the viscoelastic properties and prognostic factors associated with three main tumor types in dogs - lymphoma, carcinoma (mammary and non-mammary), and sarcoma (osteosarcoma (OSA), soft tissue sarcoma (STS), and HSA).

## 2 The pathophysiology of hemostatic abnormalities in tumor-bearing dogs

Multiple, interrelated mechanisms contribute to a prothrombotic state in tumor-bearing dogs. These mechanisms include excess procoagulant expression (20–22); inflammation and tumor-host leukocyte interactions (23–27); thrombocytosis and platelet hyper-reactivity (28, 29); turbulent tumor vasculature; consumption or inhibition of anticoagulant pathways (30); and abnormal fibrinolysis (31).

### 2.1 Excess procoagulant expression

Excess expression of procoagulant tissue factor (TF) on the surface of tumor cells, as well as TF microparticle shedding, directly activates the extrinsic pathway of the coagulation cascade (20–22, 32, 33). Tissue factor expression has previously been reported in canine mammary, lung, pancreatic, and prostatic tumors as well as fibrosarcoma and, to a lesser extent, OSA (32). In addition, increased procoagulant microparticles and the expression of externalized procoagulant phosphatidylserine on the surfaces of platelets and tumor cells provide a critical surface for the assembly of key coagulation factor complexes, such as the tenase and prothrombinase complexes (34–36).

### 2.2 Inflammation

Inflammation in tumor-bearing dogs is driven by immune targeting of tumor cells, tumor cell apoptosis, tumor necrosis due to lack of blood supply, tumor ulceration, tumor and host-derived cytokines, and upregulation of COX-2 (37–39). Inflammatory mediators such as interleukin (IL)-1, IL-6, transforming growth factor-β, and tumor necrosis factor are released by leukocytes with a subsequent increase in acute-phase proteins and procoagulants, resulting in elevated fibrinogen concentration, thrombocytosis, and increased clotting factor production (23–27). Clinical evidence of inflammation is evidenced by elevated C-reactive protein and serum amyloid A in dogs with various tumor types (6, 40). Neutrophil extracellular traps are web-like structures of decondensed DNA, histones, and antimicrobial proteins released by activated neutrophils and platelets during an immune response and are elevated in a multitude of human diseases associated with increased thrombotic risk, including cancer (41). Circulating nucleosomes are increased in dogs with a range of cancers, including lymphoma, hemangiosarcoma, histiocytic sarcoma, melanoma, and carcinoma, and may reflect cell death or leukocyte activation but do not distinguish between nucleosomes derived from apoptosis, necrosis, or neutrophil extracellular trap (NET) formation (42). NETs have been directly demonstrated in canine mammary tumors using citrullinated histone H3 and myeloperoxidase co-localization, although their functional and prognostic significance remains unclear (43). Neutrophil extracellular traps provide a scaffold for platelets and TF and Factor (F) XII activation, and are linked to VTE and poor outcomes in humans (44).

### 2.3 Thrombocytosis

In dogs with thrombocytosis, neoplasia was the second most diagnosed etiology, with an incidence of 21%−44% (45, 46). Thrombocytosis in tumor-bearing dogs is thought to be secondary to one or a combination of tumor-driven inflammation, necrosis, infection, chronic gastrointestinal blood loss, or concurrent comorbidities, although distinguishing tumor-related from non-neoplastic contributors is not always possible (28, 29, 45). In one study, 19% of dogs with carcinoma had thrombocytosis, and thrombopoietin was significantly higher than in controls, while IL-6 was not consistently different between groups, implying inflammation is not the sole driver for thrombocytosis (47). Canine OSA and mammary carcinoma cells have been reported to activate canine platelets through the P2Y12 receptor *in vitro* and contribute to platelet hyper-reactivity and subsequent platelet aggregation (48). Additionally, podoplanin, expressed in many types of canine tumors, binds to and activates platelet receptor CLEC-2, triggering signaling pathways resulting in platelet aggregation and initiation of the coagulation cascade (49).

### 2.4 Tumor vasculature

The prothrombotic microenvironment that exists within tumor vasculature is in part due to the turbulent tumor vasculature, as evidenced in 62 tumor-bearing dogs in which the majority of microthrombi identified (83%) were within the tumor vasculature (6). Aberrant angiogenesis driven by inflammatory cytokines IL-6, tumor necrosis factor, and IL-1, and subsequent vascular endothelial growth factor, results in neovascular channels characterized by high shear gradients, vascular eddies, turbulence, sluggish blood flow, and incomplete endothelial coverage, facilitating thrombosis and embolization (50–52).

### 2.5 Reduced inhibitors of coagulation

Consumption of coagulation inhibitors, such as antithrombin, an essential inhibitor of thrombin and FX activity, is evident in primary carcinoma, sarcoma, and splenic masses, in which AT activity is significantly lower than in healthy dogs (6, 53, 54). Low antithrombin activity was associated with a higher risk of mortality among dogs with neoplasia (30). Inflammation and pro-inflammatory cytokines, such as tumor necrosis factor and IL-1, can downregulate thrombomodulin, a receptor on endothelial cells that helps activate Protein C and S, both of which are essential for anticoagulation and fibrinolysis (55). Additionally, decreased protein C has been reported in dogs with metastasizing mammary carcinoma and DIC (56).

### 2.6 Altered fibrinolysis

As part of physiological hemostasis, cross-linked fibrin is formed, which the fibrinolytic system breaks down by activating tPA or urokinase plasminogen activator, converting plasminogen to plasmin. tPA is released from endothelial cells, and urokinase plasminogen activator is released from macrophages, urinary epithelial cells, or tumor cells (57). Fibrinolysis is locally regulated by PAI-1, which is released from the endothelium in response to inflammation, thrombin, and hypoxia. In cancer, increased fibrinolysis may be either primary or secondary. Primary fibrinolysis is characterized by inappropriate plasmin activation with degradation of fibrin in the absence of preceding clot formation. Primary hyperfibrinolysis has only rarely been documented in tumor-bearing dogs. In one prospective study of dogs with sarcoma, some with clinical bleeding, tPA-TEG LY30 and LY60 confirmed significant primary hyperfibrinolysis (31). Secondary fibrinolysis occurs after coagulation activation and fibrin clot formation, resulting in breakdown of cross-linked fibrin. Secondary hyperfibrinolysis is commonly reported in canine cancer patients as part of non-overt and overt DIC with associated laboratory abnormalities and/or clinical bleeding diathesis (4, 6, 8, 10, 56). In humans, clinical studies have reported that increased plasma levels of urokinase plasminogen activator are associated with cancer progression in some cancers, e.g., bladder cancer (57). This has yet to be investigated in tumor-bearing dogs.

The tumor- and host-driven alterations described above create a dynamic prothrombotic environment that is reinforced by ongoing inflammation and vascular–cellular interactions within and beyond the tumor microenvironment. The net result is that many tumor-bearing dogs exist in a state of chronic, often subclinical hemostatic alterations with occasional tipping points into overt clinical thrombosis or bleeding. In addition, hemostatic dysregulation can encourage tumor growth and metastasis by facilitating cancer cell survival in the circulation and implantation at distant sites (58–60).

## 3 Lymphoma

Studies of canine lymphoma typically focus on multicentric lymphoma, most of which report stage III-V and B-cell lymphoma (Supplementary Table 1). Lymphoma and related lymphoproliferative disorders in dogs can induce hemostatic abnormalities; however, clinically apparent macro-thrombosis and overt DIC appear less common than in dogs with carcinoma or sarcoma.

### 3.1 Thromboemboli in canine lymphoma

The prevalence of thromboembolism in dogs with lymphoma, as reported in retrospective studies evaluating the association of TED and various underlying causes, was reported as 16%, 13%, 3%−9%, and 5% in the splenic vein, pulmonary vein, portal vein, and aorta in dogs, respectively (11–13, 15, 16). Many dogs had coexisting conditions that could have contributed to the development of TED, and these studies could only report associations, not causation, between cancer and TED. Individual case reports also identify TED in the pulmonary arteries, basilar artery, and cerebral intraparenchymal vein (61–63). In a retrospective evaluation of a pathology database, intra-tumoral microthrombi were identified in 0.9% (8/872) of canine lymphoma cases; however, no thrombi distant from the primary tumor were reported on post-mortem examination following complete autopsies (64).

### 3.2 Hemostasis

#### 3.2.1 Primary hemostasis

Platelet count abnormalities in canine lymphoma are variably reported, with thrombocytopenia being the most commonly described finding, occurring in 18% (21/48), 26% (31/120), and 36% (21/57) of dogs across studies (65–67). In a study of 170 dogs with lymphoma, the median platelet count (237 x 10^3^/μl) was significantly lower compared to 170 dogs affected by other diseases, but still within the laboratory reference interval (180–451 x 103/μl) (7). The same study reported that stage V lymphoma was associated with a significantly lower platelet count than dogs with stage II-IV lymphoma and dogs affected by other diseases. Thrombocytopenia associated with advanced lymphoma is likely multifactorial, including decreased platelet production secondary to myelodysplasia, immune-mediated platelet consumption, and platelet sequestration (7). In contrast, two retrospective studies evaluating the etiology of thrombocytosis identified lymphoma as a common underlying tumor in 27% (190/699) and 14% (10/69) of dogs (45, 68). In a study of 17 dogs receiving chemotherapy (CHOP with l-asparaginase), the median platelet count at diagnosis was within the reference interval, increased significantly in the 2nd and 3rd weeks of treatment, remained within the reference interval, and decreased to baseline at week 4 (69).

Electronic impedance aggregometry using the agonists platelet-activating factor, adenosine diphosphate (ADP), and collagen, in 15 dogs with multicentric lymphoma reported a significant increase in maximum aggregation amplitude in lymphoma patients compared to healthy controls across all agonists, suggesting a lymphoma-associated platelet hyperactivation (70). In 17 dogs with lymphoma, global platelet function (capillary bleeding time, automated platelet function analysis, and maximum aggregation) remained unchanged from baseline during the first 4 weeks of CHOP chemotherapy with L-asparaginase (69). In contrast, a significant reduction in maximum aggregation amplitude for agonists ADP, collagen, and arachidonic acid using platelet turbidimetric aggregometry in seven chemotherapy-induced remission lymphoma dogs performed immediately before and 1 h after vincristine administration suggests vincristine may temporarily inhibit platelet function in lymphoma patients (71). In 66 dogs with lymphoma, no clear evidence of clinically important dysfunction based on whole-blood platelet aggregometry using ADP and AA was identified (72).

#### 3.2.2 Secondary hemostasis

Alterations of secondary hemostasis in dogs with lymphoma, as assessed by conventional plasma-based assays, are infrequent. In 10 dogs with lymphoma, in which all PT, 80% of aPTT, and 90% of fibrinogen concentration were within reference range. In the same study, dogs with non-mammary carcinoma were reported to have significantly prolonged aPTT and higher fibrinogen concentrations than dogs with lymphoma (9). Further evidence of mild secondary hemostatic changes in 10 dogs with lymphoma compared to healthy controls showed no significant difference in PT and aPTT, although AT activity was significantly decreased in lymphoma dogs compared to controls (73). In another study, 99% (96/97) of dogs with lymphoma did not have significantly abnormal PT and aPTT, with hyperfibrinogenemia reported in 28% (27/97) of dogs (66). In subtle contrast, median PT, aPTT, and fibrinogen concentrations fell within reference intervals in 89% (24/27) of PT, 67% (18/27) of aPTT, and 44% (12/27) of fibrinogen concentration measured (74). Another study reported significantly prolonged prothrombin time (PT) and thrombin time (TT) and lower fibrinogen concentration in dogs with various grades of multicentric lymphoma compared to sick dogs without lymphoma (7). No significant differences were observed between lymphoma subtypes (B or T cell), but stage V lymphoma was associated with significantly prolonged PT, increased FDPs, and decreased AT activity. Supporting the association with advanced-stage disease, 4 of 5 dogs with stage IV or V lymphoma in one study fulfilled criteria for overt DIC, based on four or more abnormal hemostatic parameters (22).

#### 3.2.3 Viscoelastic testing

Viscoelastic testing in dogs with lymphoma is limited to three studies, in which 38% (3/8) (5), 60% (6/10) (9), and 63% (17/27) (74) of dogs had TEG changes indicative of hypercoagulability at initial diagnosis. A contribution of systemic inflammation to the hypercoagulable phenotype is suggested by elevated C-reactive protein concentrations documented in two studies independent of viscoelastic investigations (75, 76). One study reported that TEG-based coagulation was not significantly different from dogs with other tumor types, including sarcomas and mammary carcinoma, but showed significantly lower MA compared to non-mammary carcinomas. In the same three studies, 10% (1/10), 30% (8/27), and 13% (1/8) of dogs had TEGs indicative of hypocoagulability. Kol et al. followed dogs up to 4 weeks after clinical remission, which was defined as occurring 1 month after cessation of all chemotherapy and 6 weeks after discontinuation of prednisone, and although 12 of the 27 dogs did not complete the study, the hypercoagulable state (increased TEG MA above the reference interval) persisted in 80% (12/15) of dogs after remission was achieved (74).

#### 3.2.4 Fibrinolysis

While D-dimer concentrations were reported as abnormal in as few as 10% (1/10) in one study when all lymphoma stages were considered, based on other studies, a potential hyper-fibrinolytic state may be present in advanced lymphoma (9). One study of 27 dogs with 78% of dogs with stage IV or V lymphoma at presentation, increased thrombin-antithrombin complexes and D-dimer concentrations were present in 85% and 55% (15/27) of dogs, respectively (74). Additionally, elevated D-dimer concentrations have been reported in 44% (21/48) of dogs at diagnosis in another study, in which 79% (38/48) had stage IV or V lymphoma (77). While D-dimer concentrations were not significantly higher in 170 dogs with various grades of multicentric lymphoma compared to 170 dogs diagnosed with other diseases, in the same study, dogs with stage V multicentric lymphoma had significantly higher FDP concentrations, hypothesized to result from primary hyperfibrinolysis in the absence of overt DIC (7). A hyperfibrinolytic state could manifest as a bleeding tendency in some advanced lymphoma cases, with thrombocytopenia compounding the risk.

#### 3.2.5 Prognostic indicators

Thrombocytopenia at diagnosis is a heterogeneous prognostic indicator, with different studies identifying a positive (78), negative (76, 79), or of no prognostic value (67, 80). One study showed that improvement or normalization of thrombocytopenia during chemotherapy is associated with longer survival (76). Dogs with intermediate to high-grade lymphoma and pretreatment plasma D-dimer concentration >500ng/ml had a significantly reduced progression-free survival and reduced overall survival time (77). Persistent hypercoagulability, specifically shortened TEG K and increased TEG angle, after chemotherapy was associated with decreased overall survival time (74).

#### 3.2.6 Conclusion

In canine lymphoma, evidence of TED is largely limited to retrospective studies of mixed-disease populations and individual case reports; therefore, the true lymphoma-specific prevalence of TED remains unknown. At diagnosis, primary hemostatic abnormalities are generally limited, with thrombocytopenia most common in advanced (stage V) disease and median platelet counts typically within reference intervals in less advanced disease. There is no convincing evidence of clinically relevant platelet dysfunction, although impedance aggregometry suggests vincristine may transiently impair platelet aggregation. Screening tests of secondary hemostasis (PT, aPTT, fibrinogen) are often within reference intervals. A proportion of dogs exhibit hypercoagulable viscoelastic profiles, potentially linked to inflammation, and increased markers of thrombin generation and fibrinolysis. Some dogs with advanced lymphoma exhibit low fibrinogen concentrations and increased FDPs, consistent with hyperfibrinolysis or DIC. These findings support a dysregulated hemostatic state in higher-stage disease with the potential for both thrombosis and bleeding. During and after chemotherapy, platelet count and function, as well as viscoelastic variables, may remain abnormal (74). Elevated D-dimer concentration at diagnosis and persistent hypercoagulability in remission have been associated with poorer outcomes. Clinically, dogs with advanced-stage lymphoma are candidates for monitoring for both TED and bleeding, with consideration of serial assessment of platelet count, coagulation screening tests, viscoelastic parameters, and fibrinolytic markers where available.

## 4 Carcinoma

Carcinomas are among the cancer types most strongly linked to hypercoagulability, thrombosis, and overt DIC in human medicine (81, 82), and a similar trend is observed in dogs. Studies in dogs are commonly classified as mammary, non-mammary, or various carcinomas (Supplementary Table 2), and differences will be highlighted where appropriate.

### 4.1 Thromboemboli in canine carcinoma

The prevalence of thromboembolism in dogs with carcinoma, as reported in retrospective studies evaluating the association of TED and various underlying causes, was reported as 15%, 6%, 9%−18%, and 11% in the splenic vein, pulmonary vein, portal vein, and aorta in dogs, respectively (11–13, 15, 16). Case reports describe thrombosis of the portal vein, caudal vena cava, aorto-iliac artery, and femoral artery associated with pancreatic carcinoma (83), renal tubular carcinoma (84), mammary anaplastic carcinoma (85), and malignant mammary gland (86), respectively. Finally, one dog was reported to have thrombosis of the caudal vena cava, right external iliac, right femoral vein, and the right common iliac associated with an adrenocortical carcinoma (87). Intra-tumoral microthrombi were identified in 1.6% (8/455) of canine carcinoma in a retrospective evaluation of a pathology database, and no macrothrombi distant to the primary tumor were reported on post-mortem samples (64). A prospective study of 30 dogs with carcinoma identified microscopic thrombi in 43% (13/30) of canine carcinoma cases. Of these, 69% (9/13) were intra-tumoral microthrombi, and 46% (6/13) were microthrombi in distant organs (6). Post-mortem examination of all 30 dogs identified one dog with macrothrombi of the portal vein (6).

### 4.2 Hemostasis

#### 4.2.1 Primary hemostasis

Thrombocytopenia or thrombocytosis may be identified in dogs with carcinoma. In a retrospective study of 214 tumor-bearing dogs, thrombocytopenia was identified in 28% (60/214) of dogs diagnosed with various carcinomas (with melanomas included in the carcinoma cohort) (65). The estimated relative risk of thrombocytopenia in dogs with carcinoma was not increased after excluding melanomas from the analysis. In contrast, two retrospective studies evaluating the etiology of thrombocytosis, the most common tumor to cause thrombocytosis was various carcinomas in 35% (24/69) and 52% (365/699) of dogs, and were responsible for 30% (365/1,254) of dogs diagnosed with thrombocytosis in a 5-year period (45, 68). Transitional cell carcinoma has been reported as the most common neoplastic diagnosis (35%, 247/699) associated with thrombocytosis, most likely attributed to chronic blood loss via hematuria (45). Thrombocytosis prevalence in prospective studies in dogs with various carcinomas was reported as 42% (15/32) (28), and 20% (12/63) (47). In the latter study, the median platelet count in dogs with carcinoma was significantly higher than in healthy control dogs, and urothelial carcinoma was again the most common carcinoma associated with thrombocytosis (9/12). Thrombopoietin concentrations were significantly higher in dogs with carcinoma than in controls, but were not different between dogs with carcinoma and those with or without thrombocytosis (47). Multiple factors are likely to affect thrombopoietin production, including inflammation, necrosis, tumor-related chronic blood loss, and concurrent disease, which can impact platelet number, production, and consumption in dogs with carcinoma (28, 29, 45).

Changes in platelet count in dogs with mammary carcinoma were similar to those observed in dogs with various carcinomas. In a prospective study of 60 dogs with mammary carcinoma, 8% of platelet counts were below, and 3% above the 95% interval of the control group (4). One prospective study reported thrombocytopenia in 10% (3/30) and thrombocytosis in 27% (8/30) of dogs with mammary carcinoma (88). A higher prevalence of thrombocytosis, 46% (15/32), was reported in another prospective study of dogs with mammary carcinoma, and platelet counts were significantly higher than in control dogs (89).

Studies evaluating platelet aggregation in dogs with carcinomas are limited to a mixed tumor cohort and an *in vitro* study of mammary carcinoma cells. Dogs with carcinomas, as well as other malignancies, were compared to controls using impedance aggregometry and collagen, adenosine diphosphate (ADP), and arachidonic acid as platelet activators. Platelet hyperaggregability was identified in dogs with malignancies compared to normal dogs, although carcinomas were not evaluated as a group (29). Mammary carcinoma cells *in vitro* activate canine platelets, resulting in platelet aggregation, and the platelet ADP receptor P2Y12 appears essential in cancer-induced platelet activation *in vivo* (48).

#### 4.2.2 Secondary hemostasis

No significant difference in PT and aPTT in dogs with various carcinomas compared to controls was reported in a prospective study, despite significant hypercoagulability based on TEG-derived thrombin generation (TEG_TG_) and hyperfibrinogenemia in 32% (9/32) of dogs with carcinoma, compared to control dogs (28). In contrast, dogs with thrombocytopenia and various carcinomas were reported to have prolonged aPTT in 43% (9/21), hypofibrinogenemia in 27% (6/21), and only infrequent prolongation of PT in 5% (1/21) (65). In a prospective study comparing various cancer types, non-mammary carcinomas showed significantly prolonged aPTT and increased fibrinogen concentration compared to mastocytomas and lymphomas, but no difference when compared to mammary carcinomas, OSAs, and STSs (9). A prospective clinical histopathology-based study identified no significant difference in fibrinogen concentration, FX, FVII, or AT activity in dogs with various carcinomas compared to dogs with sarcomas (6). When carcinomas and sarcomas were evaluated as a cohort, the tumor-bearing group showed higher fibrinogen concentration and lower FX and AT activity than healthy controls.

In dogs with mammary tumors, TF expression was identified in 44% of benign and 58% of malignant tumors, where TF acts as the direct activator of the extrinsic pathway of coagulation (20). One or more abnormal coagulation tests were reported in 67% (40/60) of dogs with mammary carcinoma, and the likelihood of hemostatic abnormalities such as prolonged aPTT, increased fibrinogen concentration, and decreased AT activity increased in advanced (stage IV) disease (4). Inflammatory tumors, extensive necrosis, tumors fixed to deeper tissues, or tumors with distant metastases were associated with more frequent hemostatic derangements (4). Three prospective studies identified higher fibrinogen concentrations in 23% (14/60) (4), 67% (16/24) (90), and 66% (21/32) (89) of dogs with mammary carcinoma, with significantly higher concentrations in clinical stage III-V compared with the control group (90), and stage IV carcinoma than stage I and II carcinoma (89). Elevated PT or aPTT was reported in 20% (18/90) (4) and 63% (20/32) (89) of dogs with mammary carcinoma, and both studies identified that aPTT was significantly prolonged in stage IV carcinoma compared to stage II.

#### 4.2.3 Viscoelastic testing

The viscoelastic evaluation of carcinomas as a cohort remains limited. A prospective study of dogs with various carcinomas reported that TEG_TG_ was significantly higher than in healthy controls, with 46% (15/32) of TEG_TG_ values exceeding the upper limit of the TEG_TG_ reference intervals for healthy age-matched dogs (28). The TEG parameters indicative of hypercoagulability compared to healthy controls for carcinoma dogs included shorter K, increased angle, and higher MA and G. A prospective study evaluated various cancer types and identified all nine dogs with non-mammary carcinomas as hypercoagulable based on TEG G above the reference interval, and significantly more hypercoagulable than lymphoma, but not other cancer types (9). A retrospective study evaluated adrenal tumors and reported 67% (6/9) of dogs with adrenocortical adenocarcinoma were hypercoagulable based on TEG G (91). Grouping carcinomas with other tumor types confounds the interpretation of hemostatic status for individual carcinoma types (5, 92). The prospective evaluation of 10 hepatic tumors, six hepatocellular carcinomas with four hepatocellular adenomas, identified a hypercoagulable state based on TEG G pre-operatively, that persisted 10–14 days post-operatively (93). Finally, dogs with various carcinomas had no kaolin-activated TEG variables significantly different from sarcomas, and when grouped with sarcomas, all variables except TEG clot initiation (R time) were indicative of hypercoagulability, and the cohort of dogs was in a state of non-overt DIC compared to healthy age-controlled dogs (6, 94).

#### 4.2.4 Fibrinolysis

Evidence of clinically relevant hyperfibrinolysis in dogs with carcinoma is sparse, although clinicopathological abnormalities have been described. D-dimer concentration was increased in 59% (10/17) of dogs with various carcinomas in a study evaluating D-dimers in various tumors (95). A protein essential in reducing fibrinolysis, plasminogen activator inhibitor-1 (PAI-1) activity, was significantly lower in dogs with various carcinomas than that in healthy dogs (15.7 vs. 26.2 IU/ml) (28). D-dimer concentrations decreased after surgical removal of various epithelial and mesenchymal tumors, supporting tumor-associated activation of coagulation and fibrinolysis (96). No difference in D-dimers or plasminogen concentration was identified between dogs with mammary carcinoma, non-mammary carcinoma, lymphoma, STS, OSA, and mastocytoma (9). In dogs with various carcinomas, no significant difference in D-dimer concentration or TEG-based lysis % at 30 and 60 min was observed compared with sarcomas. When grouped with sarcomas, both D-Dimer concentration and TEG lysis % at 60 min were significantly elevated compared to controls (6).

FDPs, secondary fibrinolysis markers, were elevated in only 7% (4/60) of dogs with mammary carcinomas (4). In contrast, in a series of seven dogs with extensive metastasizing mammary carcinoma and hemorrhagic diathesis, all dogs had markedly increased FDPs and markedly decreased plasminogen, consistent with hyperfibrinolysis and DIC (56). Hyperfibrinolysis and DIC has also been reported in a dog with metastatic nasal adenocarcinoma (97). Overall, clinically significant hyperfibrinolysis is not consistently demonstrated across carcinomas. While there is direct evidence of hyperfibrinolysis in individual canine carcinoma cases, the frequent increases in D-dimer concentration and decreases in PAI-1 activity suggest tumor-associated fibrinolysis; larger cohorts of dogs are required to provide solid evidence of hyperfibrinolysis in dogs with carcinoma.

#### 4.2.5 Prognosis

In one study of 32 dogs with various carcinomas, none of the hemostasis variables measured (PT, aPTT, fibrinogen, TEG_TG_, PAI-1, or thrombin-antithrombin complexes) was associated with metastasis (28). TEG variables have typically not been shown to identify metastatic disease, although, given the inclusion of various cancer types, their true ability to do so in carcinoma remains unknown (5, 6, 9). D-dimer concentration was significantly higher in tumor-bearing dogs with microthrombi than in those without microthrombi, suggesting increased secondary fibrinolysis that may contribute to metastatic disease; however, the group included carcinomas and sarcomas (6). In the same cohort of dogs, only TEG lysis % at 30 and 60 min (Ly60) was significantly lower in tumor-bearing dogs with metastasis compared to dogs without metastasis (98). In contrast to lower TEG-based lysis, increased circulating levels of urokinase-type plasminogen activator, a key enzyme in the conversion of plasminogen to plasmin and fibrinolysis, are observed in dogs with metastatic disease of various tumor types, including carcinomas (99). Hyperfibrinogenemia and prolonged aPTT were associated with increasing clinical stage in two studies of dogs with mammary carcinomas, but it is unclear whether these variables are directly correlated with prognosis (4, 89).

#### 4.2.6 Conclusion

Canine carcinomas are associated with a spectrum of hemostatic abnormalities, with TED frequently reported. Retrospective data and prospective necropsy studies report both macrothrombi and intra-tumoral and distant microthrombi, supportive of a state of non-overt DIC in many dogs with carcinoma. Reactive thrombocytosis is commonly present, but occasionally thrombocytopenia, with increased thrombopoietin and inflammatory or blood-loss–related drivers. A pro-aggregatory interaction between carcinoma cells and platelets has also been reported. Both mammary and non-mammary carcinomas have been reported to have hyperfibrinogenemia and, in advanced stages, prolongation of PT/aPTT and decreased antithrombin. Viscoelastic studies consistently demonstrate carcinoma-associated hypercoagulability (shorter K/R, increased angle, MA, and G) in a substantial proportion of dogs, and, when carcinomas are grouped with sarcomas, the overall profile is consistent with hypercoagulability compatible with non-overt DIC. Evidence for clinically relevant hyperfibrinolysis in carcinomas is largely confined to biomarker patterns (elevated D-dimer concentrations, reduced PAI-1) and a small number of cases showing overt DIC with marked secondary hyperfibrinolysis, suggesting that overt hyperfibrinolysis is probably uncommon. Prognostically, more advanced tumor stage, inflammatory disease, and certain hemostatic changes are associated with more aggressive carcinomas, but robust, carcinoma-specific prognostic markers based on hemostasis remain to be defined. Overall, carcinoma-associated subclinical hemostatic dysfunction is common and may be of pathobiological and clinical relevance, with implications for thrombotic risk, bleeding, and potentially metastasis and survival. It remains unclear whether treatment for hemostatic alterations should be undertaken; however, early recognition and treatment may reduce thrombosis and metastasis (see later) and improve outcomes.

## 5 Sarcoma

Sarcomas are responsible for less than 10% of cancers in people, and studies have identified the overall incidence of venous thromboemboli in sarcoma patients as 8% (100), with a range of 6.7% for Ewing sarcoma to 12% for OSA in patients (101). For this review, hemostatic changes associated with STS and OSA will be considered independent of HSA due to the specific hemostatic profile associated with canine HSA (Supplementary Table 3).

### 5.1 Thromboemboli in canine sarcoma

The prevalence of thromboembolism in dogs with sarcoma, as reported in retrospective studies evaluating the association of TED and various underlying causes, was 5, 6, and 16% in the splenic vein, portal vein, and aorta, respectively (11, 13, 15). Microthrombi formation in dogs with sarcomas is more common than in lymphoma or carcinoma. Intra-tumoral microthrombi were identified in 5% (37/722) of canine sarcomas in a retrospective evaluation of a pathology database, with no macrothrombi distant to the primary tumor reported on post-mortem samples (64). A prospective study of 32 dogs with sarcoma and post-mortem examination of all dogs identified 6% (2/32) with macrothrombi (both soft-tissue sarcomas of muscle) in the portal vein and aorta, and in the splenic vein (6). Microscopic thrombi were identified in approximately 56% (18/32) of canine sarcoma cases; of these, 94% (17/18) were intra-tumoral microthrombi, and 22% (4/18) of microthrombi were also identified in distant organs (6). HSA in various locations accounted for 61% (11/18) and OSA for 17% (3/18) of all microthrombi associated with sarcomas (6).

### 5.2 Hemostasis

#### 5.2.1 Soft tissue sarcomas and osteosarcomas

##### 5.2.1.1 Primary hemostasis

Mild and infrequent platelet changes have been reported in both STS and OSA. Abdominal visceral STS were retrospectively evaluated with thrombocytopenia in 5% (2/42) and thrombocytosis in 7% (3/42) of dogs reported (102). Two retrospective studies identified OSA as the etiology of thrombocytopenia in 5% (10/214) (65) and 4% (3/69) of dogs with tumors (68). Two retrospective studies identified mild thrombocytopenia in 20% (12/59) and 1.7% (1/66) of appendicular OSA cases, and mild thrombocytosis in 12% (7/59) and 10% (6/60) of cases (103, 104). No significant difference in median platelet count was identified in two prospective studies when various carcinomas were compared to various sarcomas, as well as when OSA and STSs were compared to a range of other tumor types (6, 9).

Three OSA cell lines (OSCA-8, OSCA-40, and OSCA-78) have been reported to activate canine platelets, and as with carcinoma cells *in vitro*, OSA cells can directly trigger P2Y12-dependent platelet aggregation (48). *In vivo* platelet aggregation studies in OSA and STS are lacking. One study, as mentioned for carcinoma, evaluated sarcomas with other malignancies, and showed platelet hyperaggregability is present in dogs with malignancies compared to normal dogs, but STS and OSA were not evaluated as a group (29).

##### 5.2.1.2 Secondary hemostasis and viscoelastic testing

In contrast to carcinoma, the evaluation of secondary hemostasis and viscoelastic data in STS and OSA is largely limited to mixed-tumor cohorts. Only two studies evaluated sarcoma-bearing dogs as a distinct cohort from other tumor types. In the first prospective observational study, neither hemostatic variables (PT, aPTT, fibrinogen, AT) nor TF-activated TEG-G differed significantly between the STS (*n* = 13) and OSA (*n* = 6) cohorts and other tumor-bearing dogs (9). The second prospective observational study also reported no significant difference in hemostatic variables (fibrinogen, FV, FVII, AT) and any kaolin-activated TEG variables between dogs with various non-hemorrhagic sarcomas (including HSA) and carcinomas (6). As previously mentioned, the tumor-bearing dogs in the latter study were significantly more hypercoagulable, based on changes in two or more TEG variables than control dogs, and had significantly higher fibrinogen concentration and significantly lower FX, FVII, and AT activity. Sarcoma cohorts that include STS and OSA have, similar to carcinomas, been associated with hypercoagulable prothrombotic viscoelastic profiles, and sarcoma-bearing dogs have been reported to be in a state of non-overt DIC compared to healthy dogs (6, 31).

##### 5.2.1.3 Fibrinolysis

D-dimer concentrations decreased after surgical removal of various mesenchymal and epithelial tumors, supporting tumor-associated activation of coagulation and fibrinolysis (96). D-dimer concentration and plasminogen activity did not differ between STS and OSA, or between other tumor cohorts, in a prospective study (9). Additionally, no difference in D-dimer concentration was observed between sarcomas and carcinomas (6). In a subset of nine sarcoma-bearing dogs considered normo-coagulable based on TF-activated TEG, primary hyperfibrinolysis was demonstrated based on tPA-modified TEG (31). This finding suggests that some sarcomas can induce significant fibrinolysis, and tPA-modified viscoelastic testing can reveal a hyperfibrinolytic tendency but does not necessarily predict spontaneous clinical bleeding. Further studies are needed to investigate potential implications for the risk of perioperative bleeding and the use of targeted antifibrinolytic therapy.

##### 5.2.1.4 Prognosis

To date, no dedicated STS studies have shown that platelet count, thrombocytosis, PT/aPTT, fibrinogen concentration, AT activity, or TEG/ROTEM parameters independently predict survival or disease-free interval. As described for carcinomas, TEG lysis % at 30 and 60 min (Ly60) were significantly lower in tumor-bearing dogs with metastasis compared to dogs without metastasis, but the cohort included carcinomas and sarcomas, and was not powered to evaluate STS and OSA separately (98).

In a retrospective study of 59 dogs with appendicular OSA treated with amputation and adjuvant chemotherapy, lower platelet count and lower platelet-crit at diagnosis were significantly associated with shorter progression-free survival in univariable analyses, but did not persist as independent predictors of progression-free survival in multivariable analysis (103). Of 60 dogs with appendicular OSA retrospectively evaluated, an elevated platelet-to-albumin ratio was significantly associated with decreased progression-free interval, asserting the interrelationship between inflammation and coagulation (104). Platelets appear to modulate tumor-platelet interaction and inhibit OSA cell migration, and there is evidence of bidirectional interaction in which tumor cells activate platelets, and platelets in turn modulate OSA cell biology and metastasis (105).

##### 5.2.1.5 Conclusion

Changes in platelet number and function in dogs with STS and OSA appear mild and subclinical. Although further studies with larger sample sizes evaluating specific STS and OSA cohorts are required, available evidence supports systemic coagulation activation with frequent intra-tumoral microthrombi and a hypercoagulable phenotype consistent with non-overt DIC. In contrast, macrothromboemboli appear to occur in a smaller subset of cases. Further studies are necessary to determine the clinical relevance and implications of these changes, and to assess whether sarcoma patients identified as hypercoagulable by viscoelastic testing are candidates for anticoagulant therapy to mitigate the risk of thrombosis and potentially reduce the incidence of metastasis (see later).

#### 5.2.2 Hemangiosarcoma

HSA has a well-established association with coagulopathies and DIC. The pathophysiology of hemostatic dysfunction in canine HSA involves tumor expression of TF, endothelial disruption with widespread intravascular fibrin deposition leading to microangiopathic changes, turbulent vasculature, and platelet consumption (8, 33).

##### 5.2.2.1 Primary hemostasis

The predominant platelet change in HSA is moderate-to-marked thrombocytopenia, which can be observed even in the absence of hemoperitoneum (8). Thrombocytopenia has been reported in 75% (18/24) of dogs with HSA of various locations, of which 50% (12/24) were diagnosed with overt DIC (8). In a retrospective study evaluating the etiology of thrombocytopenia in dogs, moderate to marked thrombocytopenia was present in 47% (18/38) of dogs with HSA, and dogs with HSA had an estimated relative risk of 8.3 (CI_95%_: 4.2, 16.9) for developing thrombocytopenia (65). In dogs with splenic HSA, 71% (50/70) had thrombocytopenia (106), and dogs with splenic HSA had significantly lower median platelet count compared to dogs with other splenic masses (107). Platelet aggregation in dogs with exclusively HSA has been evaluated in only one study, which did not demonstrate consistent depression of whole-blood aggregometry responses to ADP and AA, and apparent group differences in ATP secretion were confounded by marked baseline imbalance and a small sample size (72). No conclusion can be made regarding platelet aggregation in dogs with HSA.

##### 5.2.2.2 Secondary hemostasis and viscoelastic testing

Hemostatic dysfunction in dogs with HSA is dependent on the stage or progression of HSA but is most often associated with an initial hypercoagulable state, and can be followed by clinically relevant thrombosis, hemorrhage, and overt DIC (6). Splenic masses are often evaluated as a group that includes HSA, which confounds the interpretation of hemostasis variables and viscoelastic changes by HSA stage. Within the non-hemorrhagic splenic mass cohort, a hypercoagulable state is most commonly identified, consistent with non-overt DIC (54, 108). The study-defined criteria for overt DIC were met in 50% (12/24) of dogs with HSA of various locations at presentation in a retrospective study (8). Coagulation abnormalities included prolonged PT or aPTT, increased fibrinogen degradation products, and decreased AT activity. In a study evaluating the etiology of DIC, 35% (7/20) of dogs with DIC had HSA, with the incidence of DIC in dogs with HSA reported as 47% (7/15) (53). In a study of dogs with spontaneous hemoperitoneum, 71% (20/28) of dogs were diagnosed with HSA and evidence of hypocoagulability (decreased MA on TF-activated TEG), and evidence of fibrinolysis (109). In accordance with the latter study, a recent prospective study identified overt DIC with intracavitary hemorrhage in 16% (10/62) of dogs with sarcomas or carcinomas, 80% (8/10) of those identified with overt DIC were diagnosed with HSA in various locations (6). In the same population, dogs with overt DIC did not show significant hypocoagulability based on any kaolin-activated TEG variable compared with a healthy control group, but did exhibit intracavitary hemorrhage, a significantly lower fibrinogen concentration, and decreased FX, FVI, and AT activity.

##### 5.2.2.3 Fibrinolysis

Hyperfibrinolysis in canine HSA, often associated with increased FDPs or D-dimer concentration and DIC, has been reported in HSA studies and case series (8, 110, 111). The hypocoagulability previously described in dogs with spontaneous hemoperitoneum was accompanied by a concurrent hyperfibrinolytic state, as evidenced by increased lysis % at 30 and 60 min using TF- and 50 U/ml tPA-modified TEG, and increased D-dimer concentrations (109). The identified coagulation abnormalities were associated with shock severity and protein C deficiency. In contrast, dogs with intra-cavitary hemorrhage and HSA (80%, 8/10) did not show significantly increased kaolin-activated TEG-based lysis compared to healthy dogs, but did have significantly higher D-dimer concentrations and complied with the International Society of Thrombosis and Hemostasis requirements for overt DIC (6, 94). The lack of clot lysis on standard kaolin- or TF-activated TEG is attributable to the poor sensitivity of these activators for detecting fibrinolysis; therefore, future studies evaluating tPA-modified TEG fibrinolysis are warranted.

##### 5.2.2.4 Prognosis

Early evidence highlighting the prognostic importance of hemostasis in dogs with HSA comes from a study reporting that 25% (6/24) of deaths were due to hemostatic abnormalities (8). Survival time is shortest for stage III HSA, typically reflecting euthanasia decisions in the context of metastatic disease and associated hemorrhage (112), with visceral HSA the neoplasia most commonly diagnosed (76% (51/67)) in dogs with hemoperitoneum (113). In 70 dogs diagnosed with splenic HSA, perioperative thrombocytopenia was associated with shorter progression-free interval (HR, 2.2) and overall survival time (HR, 2.0) (106); and in 63 dogs diagnosed with splenic HSA, lower platelet-lymphocyte ratio was associated with shorter disease-free interval and shorter overall survival in univariable analysis, but did not remain independently prognostic in the multivariable model (107).

##### 5.2.2.5 Conclusion

In conclusion, canine HSA is characterized by a complex, stage-dependent hemostatic dysregulation in which tumor-driven procoagulant signaling, endothelial dysfunction, platelet consumption, and dynamic shifts in coagulation and fibrinolysis produce a hypercoagulable phenotype that can progress to thrombosis, hemorrhage, and DIC. Thrombocytopenia is common and often marked, and DIC criteria are met in a large proportion of dogs at presentation. Viscoelastic testing indicates that both hypercoagulability or hypocoagulability with fibrinolysis may be present, particularly in hemoperitoneum and shock, depending on the stage of disease. Hemostatic complications contribute directly to mortality and appear to correlate with outcomes, with perioperative thrombocytopenia and related indices associated with shorter survival in splenic HSA cohorts. However, interpretation of prognostic associations in dogs with HSA must consider euthanasia bias as a confounding factor, as clinical decision-making and discussions with owners—particularly in the context of bleeding complications—are likely to substantially influence outcome. Routine hemostatic assessment, including consideration of more sensitive fibrinolysis assays, would improve clinical recognition of thrombotic or bleeding risk and better define prognostic and therapeutic targets in dogs with HSA.

## 6 The role of platelets, fibrinogen, hypercoagulability, and microthrombi in metastasis

### 6.1 Platelets and metastasis

In human oncology, meta-analyses indicate that aspirin-based antiplatelet therapy is associated with a reduction in cancer-associated thromboembolic events, as well as evidence of a reduction in metastatic progression (114). In mice, the role of platelets in favoring hematogenous lung metastasis of melanoma cells was evaluated in platelet-deficient mice and in mice with homozygous deficiency of protease-activated receptor 4, an important platelet receptor activated by thrombin, responsible for platelet activation, aggregation, and thrombus formation (115). Altered platelet count and function in mice conferred a marked protective effect against melanoma metastasis, suggesting that platelets and thrombin activation can contribute to hematogenous metastasis via both thrombin-dependent and thrombin-independent mechanisms. Additionally, platelets have been shown to promote metastasis in carcinoma and melanoma by binding to circulating tumor cells, allowing protection from shear forces and immune attack, assisting in tumor cell attachment to endothelium, and establishing a metastatic niche (116). In contrast, in sarcomas, platelets and platelet-associated molecules inhibited the migration of three canine OSA cell lines (105), and the effect of platelets was shown to also inhibit sarcoma invasion in both murine and human *in vitro* models (116). Platelets in breast carcinoma promoted metastasis in mice, but not fibrosarcoma (116). The role of platelets in metastasis across tumor types requires further investigation in dogs.

### 6.2 Fibrinogen and metastasis

The role of fibrinogen in metastasis was first reported in fibrinogen-deficient mice injected with Lewis lung carcinoma or melanoma cells; fibrinogen deficiency reduced the number of metastases but did not affect the growth of metastatic foci (117). Subsequent work supported the role of fibrin(ogen) in metastasis by protecting circulating tumor cells from clearance by natural killer cells, thereby facilitating a pre-metastatic niche for survival, and by promoting adhesion, survival, and extravasation (60, 118–120).

### 6.3 Hypercoagulability, microthrombi and metastasis

Hypercoagulability may play a direct role in clinical outcomes and possibly metastasis of the cancer itself. Indicators of hypercoagulability include increased fibrinogen concentration secondary to inflammation and thrombin antithrombin complexes, but global assessment of hemostasis through viscoelastic testing has been particularly useful in demonstrating a hypercoagulable profile in cancer patients (5, 6, 9, 74). Hypercoagulability can directly promote metastasis through the effects of thrombin on tumor growth, as seen with *in vitro* and *in vivo* canine OSA cells (19). In both human and veterinary oncology, cancer-associated hypercoagulability is associated with the formation of microthrombi in the vasculature (6, 121, 122). Microthrombi are a meshwork of cross-linked fibrin that forms a thrombus-like structure within microscopic blood vessels, adherent to the vessel wall after tissue processing, with varying amounts of aggregated platelets, white blood cells, and red blood cells. Microthrombi represent a downstream manifestation of platelet activation and fibrin(ogen) deposition and likely contribute to areas of tumor necrosis and inflammation. In the context of platelets and fibrinogen, microthrombi facilitate metastatic progression by promoting a microenvironment for circulating tumor cells that supports immune evasion, survival, adhesion, and extravasation. A prospective post-mortem study identified microthrombi in 50% (31/62) of dogs with carcinoma or sarcoma, the majority (84%) of which were intra-tumoral, but one-third of affected dogs had microthrombi in distant sites such as the lung, liver, or kidneys (6). These microthrombi are likely a consequence of hemostatic changes associated with non-overt DIC. The bulk of cancer-associated thrombosis in dogs is microscopic and due to a lack of diagnostic possibilities are underdiagnosed antemortem. Tumor-bearing dogs with microthrombi had significantly higher D-dimer concentrations than those without microthrombi, and an elevated D-dimer concentration (>500 ng/ml) was 80% sensitive for predicting microthrombi in that cohort, though poorly specific (41%). Recognizing this hypercoagulable state, reducing platelet activation, fibrin production, and subsequent microthrombi formation could create opportunities for interventions, such as antiplatelet drugs or anticoagulants, that might reduce metastatic disease and improve patient outcomes.

## 7 The use of antithrombotics in cancers

In human oncology, thromboprophylaxis and anticoagulant therapy are now standard considerations due to the high incidence of cancer-associated thrombosis (81, 123). Guidelines have been established and it has been documented that using anticoagulants in at-risk cancer patients can reduce TED and may improve survival in some cases. Improvements in disease-free interval and survival have been reported with warfarin therapy in patients with small-cell lung carcinoma (124, 125). Low-molecular-weight heparin prolongs overall survival in various cancers (126–128), and various types of heparins can inhibit tumor cell growth and metastasis and even enhance tumor cell chemosensitivity (129). In human cancer patients a multitude of recommendations exist based on the type of cancer, predisposing conditions, current presence of VTE, hospitalized or ambulatory (e.g., Khorana score) status, and type of antithrombotic (130–132). Patients with highly thrombogenic malignancies (such as pancreatic and gastric cancers or lung carcinoma) or those hospitalized or receiving certain chemotherapies may be placed on prophylactic anticoagulants to prevent VTE (130, 133). In recent years, direct oral anticoagulants such as apixaban and rivaroxaban have emerged as effective and convenient alternatives to low-molecular-weight heparins for cancer patients, although careful attention is paid to bleeding risk, particularly in cancers of the GI tract (134). Current human guidelines typically recommend anticoagulation for at least 3–6 months for cancer patients with confirmed thrombosis (135).

**Figure 1:**
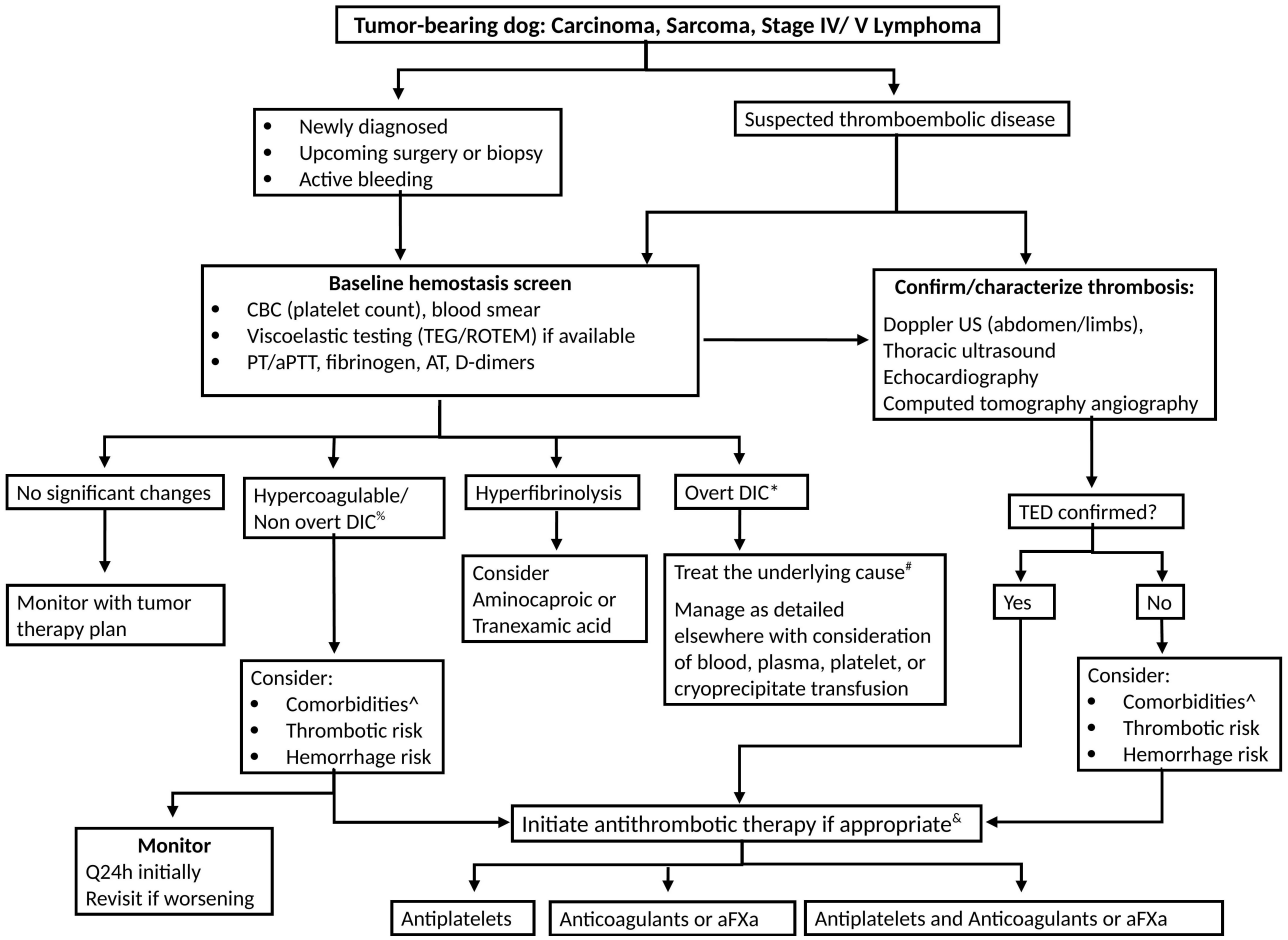
Proposed algorithm for the diagnosis and management of hemostatic dysfunction in tumor-bearing dogs. ^*^Criteria for the diagnosis of DIC include clinical bleeding and decreased fibrinogen, hypocoagulable thromboelastogram, inhibitor consumption (decreased AT activity), and increased fibrinolytic activity (increased D-dimer concentration), which are based on the International Society of Thrombosis and Hemostasis criteria, previously validated in dogs (94, 136). ^%^Criteria for the diagnosis of non-overt DIC include increased fibrinogen, procoagulant activation (decreased coagulation factor activity e.g. FV-FXII), hypercoagulable thromboelastogram, inhibitor consumption (decreased AT activity), and increased fibrinolytic activity (increased D-dimer concentration) (6, 137). ^#^Hypercoagulability is common in canine cancer; overt DIC is less common in many tumor cohorts—avoid reflex transfusion for mild to moderate hemostasis changes without bleeding. ^∧^Systemic inflammation, vascular stasis or endothelial dysfunction. ^&^Further studies are required to determine the appropriate conditions for initiating antithrombotic therapy, as well as the optimal antithrombotic therapy for each tumor type. aFXa, anti-factor Xa; aPTT, activated partial thromboplastin time; AT, antithrombin activity; DIC, disseminated intravascular coagulation; ROTEM, rotational thromboelastometry; TED, thromboembolic disease; TEG, thromboelastography; PT, prothrombin time.

In veterinary oncology, the clinical importance of laboratory hemostatic abnormalities and whether targeting them as part of the management of canine cancer patients, outside of patients with overt TED or bleeding diathesis, remains to be established. There are currently no guidelines for prophylactic antiplatelet or anticoagulation in dogs with tumors. An algorithm, based on current evidence, for identifying hemostatic dysfunction and candidates for antiplatelet or anticoagulant therapy is proposed in Figure 1. Therapy should be individualized based on patient-specific clinical or laboratory thrombotic risk factors, including concurrent comorbidities, systemic inflammation, vascular stasis, or endothelial dysfunction. With increasing evidence of the hypercoagulable state, presence of non-overt DIC, thrombotic risk, and a possible survival benefit (yet to be evaluated in dogs) associated with anticoagulation, this may change in the future. Compelling parallels to human cancer-associated hemostatic alterations raise the question of whether treating the coagulation disorder could also slow cancer progression. Tumors thrive on the prothrombotic environment, so repurposing anti-coagulation as anti-cancer therapy is an intriguing concept. The most appropriate prophylactic anticoagulants for dogs remain unknown. Anticoagulant options include clopidogrel, unfractionated heparin, low-molecular-weight heparins, and direct oral anticoagulants or FXa inhibitors. Future studies on individual cancer types (e.g., OSA, HSA, etc.) rather than cancer groups are integral to advancing this field. HSA is a unique example of the possible need for anticoagulants in the early stage of disease to prevent microthrombi, but later in the disease, the prevention of bleeding and hyperfibrinolysis may be more beneficial, and antifibrinolytic therapy with tranexamic or aminocaproic acid could be considered (8, 31). As diagnostics such as bedside viscoelastic testing become more widely available in veterinary hospitals and as studies focus on individual cancer types, stratification of canine cancer patients who would benefit most from anticoagulation may improve both patient quality of life and survival.

## 8 Conclusion

Hemostatic dysfunction arises from a complex interplay between tumor environment and the host's coagulation system – tumors can activate coagulation through a variety of ways, including TF and inflammatory pathways, leading to a spectrum of abnormalities from subclinical hypercoagulability to life-threatening TED or DIC and bleeding. A systematic approach to the identification of hemostatic dysfunction in tumor-bearing dogs is presented in this review; however, it is complicated by the challenge of acquiring advanced imaging and anesthesia to identify TED, and there are no evidence-based treatment guidelines for thromboprophylaxis.

Future research may include developing tests independent of advanced imaging to identify TED and tumor-specific thromboprophylaxis strategies that balance thrombotic prevention with hemorrhagic risk. Validated thromboprophylaxis may serve as an adjunct anticancer approach by limiting microthromboembolic metastatic progression and potentially improving survival outcomes.
