# Supplementary material for: Thromboembolic disease and hemostatic alterations in tumor-bearing dogs – A narrative review
Source: Front Vet Sci. 2026 Jun 5;13:1818630. doi: 10.3389/fvets.2026.1818630 (PMC13280929; doi:10.3389/fvets.2026.1818630)
Supplement: Supplementary file 1 [file Table_1.docx]

**Supplementary Table 1:** Number of cases, study design, anatomical location, clinical staging, immunophenotype classification, and hemostasis evaluated in canine lymphoma studies. Case numbers are indicated in parentheses.

| **Reference number** | **Reference** | **Number of lymphoma cases** | **Study design** | **Anatomical site of lymphoma** | **Stage of lymphoma** | **Immunophenotypic classification / histological classification** | **Hemostasis variables evaluated** |
| --- | --- | --- | --- | --- | --- | --- | --- |
| 5. | Kristensen et al., 2008 | 8 | Prospective cross-sectional | NR | NR | NR | Plt, PT, aPTT, D-dimer, TF-TEG |
| 7. | Messina et al., 2024 | 170 | Retrospective | Multicentric (170) | II (1), III (12), IV (49), V (108) | B-cell (91), T-cell (79) | Plt, PT, aPTT, thrombin time, fibrinogen, FDP, D-dimers, AT |
| 9. | Andreasen et al., 2012 | 10 | Prospective cross-sectional | Multicentric (8), mediastinal (1), gastrointestinal (1) | Ib (1), IIb (1), IIIb (4), IVb (3), Vb (1) | B-cell lymphoblastic (4), T-cell lymphoblastic (1), unknown immunophenotype: lymphoblastic (4), lymphocytic (1) | Plt, PT, aPTT, fibrinogen, AT, D-dimer, plasminogen, TF-TEG |
| 22. | Kobayashi et al., 2020 | 10, 10 controls | Case series case-control | Gastrointestinal (4), multicentric (2), hepato-splenic (2), renal, (1), mediastinal (1) | NR | B-cell (5), T-cell (5) | Plt, PT, aPTT, fibrinogen, AT, D-dimer, microparticle-associated tissue factor |
| 45. | Woolcock et al. 2017 | 715 with thrombocytosis; 1,430 controls, 190 with lymphoma | Retrospective case-control | NR | NR | NR | Plt |
| 64. | Pazzi et al., 2022 | 872 | Retrospective cross-sectional | NR | NR | NR | Pathological evaluation of the presence of microthrombi |
| 65. | Grindem et al. 1994 | 57 |  | NR | NR | NR | Plt, PT, aPTT, fibrinogen, FDPs |
| 66. | Gavazza et al., 2008 | 120 | Retrospective | Multicentric (114)  alimentary (2)  mediastinal (2)  skin (2) | NR | High-grade (94), Low-grade (17);  B-cell (15), T-cell (7) | Plt, PT, aPTT, fibrinogen |
| 67. | Gavazza et al., 2024 | 114, 60 controls | Retrospective case-control | Multicentric (114) | NR | Large B-cell (114) | Plt, Plt:lymphocyte ratio, Plt:neutrophil ratio, Plt volume:Plt ratio |
| 68. | Neel et al. 2012 | 165 dogs with thrombocytosis, 10 with lymphoma | Retrospective | NR | NR | NR | Plt |
| 69. | Eberle and Mischke, 2010 | 17 | Prospective cross-sectional | Multicentric (17) | IVa (5), IVb (9), Vb (3) | B-cell (16), T-cell (1) | Plt, capillary bleeding time, platelet aggregation (PFA: collagen/ epinephrine, collagen/ ADP; and Born-method) |
| 70. | Thomas & Rogers, 1999 | 15,  10 controls | Prospective cross-sectional case-control | Multicentric (15) | IIIa (6), IIIb (2), IVa (2), IVb (3), Vb (2) | Lymphoblastic (14), prolymphocytic (1) | Plt, platelet aggregation (platelet-activating factor, ADP, collagen), platelet ATP secretion |
| 71. | Grau-Bassas et al., 2000 | 7 | Prospective cross-sectional | Multicentric (6), gastrointestinal (1) | NR | NR | Plt, platelet aggregation (ADP, collagen, arachidonic acid) |
| 72. | McNiel et al., 1999 | 66 | Prospective longitudinal | NR | IIIa & IVa | NR | Plt, PT, aPTT, platelet aggregation (collagen, ADP, arachidonic acid), platelet ATP secretion |
| 73. | Rogers et al., 1992 | 10, 10 controls | Prospective longitudinal case-control | Multicentric (10) | IIIa (5), IIIb (2), IVb (1), Vb (2) | NR | Plt, PT, aPTT, AT, plasminogen, |
| 74. | Kol et al., 2013 | 27 | Prospective longitudinal | Multicentric (27) | IIIa (5), IIIb (1), IVa (5), IVb (6), Va (6), Vb (4) | B-cell (20), T-cell (6), unknown (1) | Plt, PT, aPTT, fibrinogen, D-dimer, AT, thrombin-AT, Kaolin-TEG |
| 75. | Nielsen et al., 2007 | 22 | Prospective cross- sectional | Multicentric (22) | Substage: a (14), b (8) | High grade (22)  B-cell (9), T-Cell (3) | CRP |
| 76. | Jeong et al., 2023 | 77 | Retrospective | Multicentric (67),  alimentary (6),  mediastinal (2),  cutaneous (1),  miscellaneous extranodal (1) | I (2), IIa (1), IIb (1), IIIa (5), IIIb (2), IVa (10), IVb (21), Va (10), Vb (25) | T-cell (3), B-cell (9) | Plt, CRP |
| 77. | Boye et al., 2020 | 48 | Prospective longitudinal | NR | II (2), III (8),  IV (29), V (9) | B-cell (37), T-cell (7), unclassified (4) | D-dimer |
| 78. | Huang et al., 2020 | 54 | Retrospective | Multicentric (54) | II (1), III (23), IV (24),  V (6) | B-cell (12), T-cell (1) | Plt |
| 79. | Childress et al., 2018 | 98 | Retrospective | Primary nodal diffuse large B-cell lymphoma | I (1), III (15),  IV (49), V (33) | Grade: low (37), intermediate (45), high (16) | Plt |
| 80. | Sutthigran et al., 2024 | 41 |  | Multicentric (41) | IIIa (9), IVa (21), IVb (6), Va (4), Vb (1) | NR | Plt, Plt:lymphocyte ratio, Plt:neutrophil ratio |

Abbreviations: ADP, adenosine diphosphate; aPTT, activated partial thromboplastin time; AT, antithrombin activity; ATP, Aaenosine triphosphate; CRP, C-reactive protein; FDP, fibrinogen degradation products; NR: not reported, PAI-1, plasminogen activator inhibitor-1; Plt, platelet count; PT, prothrombin time; TF, tissue factor; TEG, thromboelastography; SCC, squamous cell carcinoma.
