# Supplementary material for: Thromboembolic disease and hemostatic alterations in tumor-bearing dogs – A narrative review
Source: Front Vet Sci. 2026 Jun 5;13:1818630. doi: 10.3389/fvets.2026.1818630 (PMC13280929; doi:10.3389/fvets.2026.1818630)
Supplement: Supplementary file 2 [file Table_2.docx]

**Supplementary Table 2.** Number of cases, study design, histological classification, and hemostasis evaluated in canine carcinoma. Case numbers are indicated in parentheses.

| **Reference number** | **Reference** | **Number of cases** | **Study design** | **Histological classification** | **Hemostasis variables evaluated** |
| --- | --- | --- | --- | --- | --- |
| 4. | Stockhaus et al., 1999 | 60 mammary carcinoma-bearing dogs | Prospective cross-sectional | Mammary carcinoma (staged I–IV): adenocarcinoma (56), carcinoma (3), and SCC (1) | Plt, PT, aPTT, thrombin time, fibrinogen, FV, FVIII, FX, AT, FDP |
| 5. | Kristensen et al., 2008 | 49 tumor-bearing dogs | Prospective cross-sectional | Carcinomas (13), hemangiosarcoma (8), lymphoma (8), perianal adenoma (2), benign mammary tumors (3), others (1 each) | Plt, PT, aPTT, D-dimer, TF-TEG |
| 6. | Pazzi et al., 2023 | 62 tumor-bearing dogs: 30 carcinoma, 32 sarcoma; 20 controls | Prospective cross-sectional case-control | 3 most common carcinomas: mammary (16), pulmonary (3), hepatocellular (2), eight other carcinoma types | Plt, PT, aPTT, fibrinogen, D-dimer, FX & FVII, AT, D-dimer, kaolin-TEG |
| 9. | Andreasen et al., 2012 | 71 tumor-bearing dogs | Prospective cross-sectional | Mammary carcinoma (23), non-mammary carcinomas (9), osteosarcoma (n = 6), soft tissue sarcomas (13), mastocytoma (12), lymphoma (10) | Plt, PT, aPTT, fibrinogen, AT, D-dimer, plasminogen, TF-TEG |
| 20. | Andreasen et al., 2016 | 28 mammary tumor-bearing dogs (38 mammary tumors) | Prospective cross-sectional | Malignant mammary carcinomas (31), benign adenoma (5), benign mixed (2), hyperplasia (2) | Plt, PT, aPTT, fibrinogen, AT, D-dimer, plasminogen, TF-TEG, TF immunohistochemistry |
| 28. | Saavedra et al., 2011 | 32 carcinoma-bearing dogs; 19 controls | Prospective cross-sectional case-control | 3 most common: thyroid (9), anal sac (7), mammary gland (5), five other carcinoma types | Plt, PT, aPTT, fibrinogen, thrombin-AT complex, PAI-1-TEG, thrombin generation |
| 29. | McNiel et al., 1997 | 59 tumor-bearing dogs; 24 controls | Prospective cross-sectional case-control | 3 most common carcinomas (13): SCC (3), mammary adenocarcinoma (2), hepatocellular carcinoma (2), six other carcinoma types | Plt, platelet aggregometry (collagen, adenosine diphosphate, arachidonic acid), platelet ATP secretion |
| 45. | Woolcock et al., 2017 | 715 dogs with thrombocytosis; 1,430 controls | Retrospective | 3 most common: Transitional cell carcinoma (247), SCC: (22), nasal carcinoma (18), and nine other carcinoma types | Plt |
| 47. | Cheney et al., 2022 | 63 carcinoma-bearing dogs; 53 controls | Prospective cross-sectional case-control | 3 most common: Urothelial (30), nasal carcinoma (8), apocrine gland anal sac adenocarcinoma (4), ten other carcinoma types | Plt, interleukin-6, thrombopoietin |
| 56. | Mischke et al., 1998 | 7 tumor-bearing dogs | Case series | Infiltrative mammary carcinoma with metastases (7) | Plt, PT, aPTT, TT, fibrinogen; Factors II, V, VII, X, VIII:C, IX, XI, XII, AT, protein C, plasminogen, alpha 2-plasmin inhibitor, FDP, resonance thrombogram |
| 64. | Pazzi et al., 2022 | 455 | Retrospective cross-sectional | NR | Pathological evaluation of the presence of microthrombi |
| 65. | Grindem et al., 1994 | 2,059 tumor-bearing dogs, 214 thrombocytopenic dogs | Retrospective | Carcinomas (17): SCC (7), pulmonary carcinoma (5), nasal carcinoma (5) | Plt, PT, aPTT, fibrinogen, FDPs |
| 68. | Neel et al., 2012 | 165 dogs with thrombocytosis | Retrospective | Carcinomas (24): nasal carcinoma (4), apocrine gland adenocarcinoma (3); no further details provided | Plt |
| 88. | dos Anjos et al., 2018 | 55 tumor-bearing dogs; 10 controls | Prospective cross-sectional | Mammary carcinoma (30) evaluated as a group | Plt, PT, aPTT, fibrinogen |
| 89. | de Oliveira et al., 2019 | 32 mammary carcinoma-bearing dogs; 30 controls | Prospective cross-sectional case-control | Mammary carcinoma (staged I-IV): mixed tumor carcinoma, papillary carcinoma, solid carcinoma, tubular carcinoma, adeno-myoepithelioma malignant; numbers in subgroups not specified | Plt, PT, aPTT, fibrinogen |
| 90. | Duda et al., 2017 | 24 mammary tumor-bearing dogs | Prospective cross-sectional | Mammary carcinoma (28), mixed myoepithelial tumor (3), sarcoma (1); histologic grade II–III reported | Plt, PT, aPTT, fibrinogen, TT, D-dimer |
| 91. | Mitsui et al., 2024 | 30 dogs underwent adrenalectomy | Retrospective | Adrenocortical adenoma (10), adrenocortical adenocarcinoma (9), pheochromocytoma (8); adrenocortical hyperplasia (1), poorly differentiated sarcoma (1), both adrenocortical adenocarcinoma and pheochromocytoma (1) | Kaolin-TEG (G) |
| 92. | Bruno et al., 2022 | 11 tumor-bearing dogs | Prospective longitudinal | Thyroid carcinoma (3), one of leiomyosarcoma, hepatocellular carcinoma, liposarcoma, fibrosarcoma, pheochromocytoma, adrenocortical carcinoma, maxillary osteosarcoma, and costal chondrosarcoma | Plt, PT, aPTT, fibrinogen, ROTEM (in-TEM, ex-TEM, fib-TEM), thrombin generation |
| 93. | Fontes et al., 2023 | 10 hepatic tumor-bearing dogs; 8 control dogs | Prospective longitudinal case-control | Hepatocellular carcinoma (6) and hepatobiliary carcinoma (4) | Plt, PT, aPTT, fibrinogen, TEG |
| 95. | Torok-Nagy et al., 2020 | 50 tumor-bearing dogs; 20 controls | Prospective cross-sectional | Various carcinoma (17), sarcoma (10), mast cell tumor (6), lymphoma (5), insulinoma (3), lipoma (3), adenoma (3), other tumor types (3) | D-dimer |
| 96. | Ke et al., 2023 | 30 tumor-bearing dogs; 30 controls | Prospective longitudinal case-control | Mast cell tumor (7), mammary gland tumor (not specified – 7), melanoma (4), sarcoma (4), anal sac tumors (3), carcinoma (2), lipoma (2) | Plt, PT/aPTT, fibrinogen, D-dimer, TF-TEG |
| 97. | Granger et al., 2024 | 1 tumor-bearing dog | Case report | Metastatic nasal adenocarcinoma | Plt, PT, aPTT, fibrinogen, AT, D-dimer, FDP, Factors (II, VII, VIII, X), vWF:Ag, TEG (native, TF- & PAI-1) |
| 98. | Pazzi et al., 2026 | 62 tumor-bearing dogs: 30 carcinoma, 32 sarcoma; 20 controls | Prospective cross-sectional case-control | 3 most common carcinomas: mammary (16), pulmonary (3), hepatocellular (2), eight other carcinoma types | Plt; PT, aPTT, fibrinogen, D-dimer, FX & FVII, AT, D-dimer, kaolin-TEG |
| 99. | Ramos et al., 2017 | 20 tumor-bearing; 21 controls | Prospective cross-sectional case-control | Carcinoma (7) tumors, sarcoma (5), hematopoietic (4), mixed (3), gonadal (1) | Serum urokinase-type plasminogen activator (uPA) |

Abbreviations: aPTT, activated partial thromboplastin time; AT, antithrombin activity; FDP, fibrinogen degradation products; PAI-1, plasminogen activator inhibitor-1; Plt, platelet count; PT, prothrombin time; TF, tissue factor; TEG, thromboelastography; SCC, squamous cell carcinoma.
