# Supplementary material for: Thromboembolic disease and hemostatic alterations in tumor-bearing dogs – A narrative review
Source: Front Vet Sci. 2026 Jun 5;13:1818630. doi: 10.3389/fvets.2026.1818630 (PMC13280929; doi:10.3389/fvets.2026.1818630)
Supplement: Supplementary file 3 [file Table_3.docx]

**Supplementary Table 3.** Number of cases, study design, histological classification, and hemostasis evaluated in canine sarcoma. Case numbers are indicated in parentheses.

| **Reference number** | **Reference** | **Number of cases** | **Study design** | **Histological classification** | **Hemostasis variables evaluated** |
| --- | --- | --- | --- | --- | --- |
| 6. | Pazzi P et al., 2023 | 62 tumor-bearing dogs: 32 sarcoma, 30 carcinoma; 20 controls | Prospective cross-sectional case-control | 3 most common sarcomas: HSA (13), OSA (7), STS (6), four other sarcoma types | Plt, PT, aPTT, fibrinogen, D-dimer, FX & FVII, AT, D-dimer, kaolin-TEG |
| 8. | Hammer et al., 1991 | 24 HSA-bearing dogs | Prospective cross-sectional | Sites*: spleen (10), subcutaneous (5), right atrium (4), liver (4), omentum (1) | Plt, PT, aPTT, fibrinogen, FDP |
| 9. | Andreasen et al., 2012 | 71 tumor-bearing dogs | Prospective cross-sectional | Mammary carcinoma (23), non-mammary carcinomas (9), OSA (n = 6), STS (13), mastocytoma (12), lymphoma (10) | Plt, PT, aPTT, fibrinogen, AT, D-dimer, plasminogen, TF-TEG |
| 29. | McNiel et al., 1997 | 59 tumor-bearing dogs; 24 controls | Prospective cross-sectional case-control | 3 most common sarcomas: STS (12), OSA (5), HSA (4), two other sarcoma types | Plt, platelet aggregometry (collagen, ADP, arachidonic acid) and platelet ATP secretion |
| 31. | Langhorn et al., 2021 | 9 sarcoma-bearing dogs | Prospective cross-sectional | HSA (5), STS (3), undifferentiated sarcoma (1) | TF-TEF, tissue plasminogen activated -TEG |
| 53. | Maruyama et al., 2004 | 208 malignant tumor-bearing dogs | Retrospective | Carcinoma (57), sarcoma (23), other (126) | Plt, PT, aPTT, fibrinogen, AT, FDP |
| 54. | McPhetridge et al., 2022 | 15 dogs undergoing splenectomy | Prospective cross-sectional | Malignant: sarcomas (4), hemangiosarcoma (3), lymphoma (1); benign: hematoma (4), lymphoid hyperplasia (2), nodular hyperplasia (1) | Plt, PT, aPTT, fibrinogen, AT, TAT, von Willebrand factor activity (vWF:Ag), kaolin-TEG |
| 64. | Pazzi et al., 2022 | 722 | Retrospective cross-sectional | NR | Pathological evaluation of the presence of microthrombi |
| 65. | Grindem et al., 1994 | 2,059 tumor-bearing dogs, 214 thrombocytopenic dogs | Retrospective | HSA (18), OSA (10) | Plt, PT, aPTT, fibrinogen, FDPs |
| 68. | Neel et al., 2012 | 165 dogs with thrombocytosis | Retrospective | Sarcomas (13): HSA (3), OSA (3); no further details provided | Plt |
| 72. | McNiel et al.,1999 | 13 HSA-bearing dogs | Prospective longitudinal | Not reported | Platelet aggregometry (collagen, ADP, arachidonic acid) and platelet ATP secretion |
| 96. | Ke et al., 2023 | 30 tumor-bearing dogs; 30 controls | Prospective longitudinal case-control | Mast cell tumor (7), mammary gland tumor (not specified – 7), melanoma (4), sarcoma (4), anal sac tumors (3), carcinoma (2), lipoma (2) | Plt, PT/aPTT, fibrinogen, D-dimer, TF-TEG |
| 98. | Pazzi P et al., 2026 | 62 tumor-bearing dogs: 32 sarcoma, 30 carcinoma, 20 controls | Prospective cross-sectional case-control | 3 most common sarcomas: HSA (13), OSA (7), STS (6), four other sarcoma types | Plt, PT, aPTT, fibrinogen, D-dimer, FX & FVII, AT, D-dimer, kaolin-TEG |
| 102. | Linden et al., 2019 | 42 abdominal visceral STS-bearing dogs | Retrospective | 3 most common: leiomyosarcoma (16), STS (12), stromal sarcoma (8), three other sarcoma types | Plt |
| 103. | Rigas et al., 2023 | 59 OSA-bearing dogs | Retrospective | OSA (59) | Plt and associated indices |
| 104. | Jeffcoat et al., 2025 | 60 OSA-bearing dogs | Retrospective | OSA (60) | Plt, platelet-to-albumin ratio, platelet-to-lymphocyte ratio |
| 106. | Masyr et al., 2021 | 70 HSA-bearing dogs | Retrospective | Splenic HSA (70) | Plt |
| 107. | Marques et al., 2025 | 63 HSA-bearing dogs | Retrospective | Splenic HSA (63) | Plt, and associated indices, platelet-lymphocyte ratio |
| 108. | Phipps et al., 2020 | 34 dogs undergoing splenectomy | Prospective cross-sectional | Malignant: HSA (15), histiocytic sarcoma (1), splenic stromal sarcoma (1), T-cell lymphoma (1); Benign: hematoma (6), primary infarction (2), myelolipoma (2), and five other benign conditions | Plt, kaolin-TEG |
| 109. | Fletcher et al., 2016 | 28 dogs with spontaneous hemoperitoneum; 28 controls | Prospective cross-sectional case-control | HSA (20), carcinoma (5), histiocytic sarcoma, splenic soft tissue sarcoma, benign hematoma | Plt, PT, PTT, fibrinogen, AT, Protein C, D-dimer, TF- & tissue plasminogen activated-TEG |
| 110. | Mischke et al., 2005 | 30 HSA-bearing dogs | Prospective cross-sectional | Sites: spleen (18), spleen & liver (10), liver (2); metastasis in 9 dogs | Plt, thrombin time, fibrinogen, FDP, resonance thrombogram |
| 111. | Hargis et al., 1991 | 11 cutaneous tumor-bearing dogs | Case series | Cutaneous HSA and hemoangiomas | Plt, fibrinogen, PT, aPTT, FDP |

Abbreviations: ADP, adenosine diphosphate; aPTT, activated partial thromboplastin time; FDP, fibrinogen degradation products; HSA, hemangiosarcoma; OSA, osteosarcoma; Plt, Platelet count; PT, prothrombin time; TEG, thromboelastography; TF, tissue factor; STS, soft tissue sarcoma; *based on the first listed was the primary site.
